# Knowledge and attitudes towards TB among healthcare workers in Yogyakarta, Indonesia

**DOI:** 10.5588/pha.22.0017

**Published:** 2022-09-21

**Authors:** S. Main, B. Dwihardiani, A. Hidayat, S. Khodijah, J. Greig, G. Chan, A. E. Parry, B. Nababan, I. Billy, P. du Cros, R. Triasih

**Affiliations:** 1 Tuberculosis Elimination and Implementation Science Working Group, Burnet Institute, Melbourne, VIC, Australia; 2 National Centre for Epidemiology and Population Health, Australian National University, Canberra, ACT, Australia; 3 Centre of Tropical Medicine, Faculty of Medicine, Public Health and Nursing, Universitas Gadjah Mada, Yogyakarta, Indonesia; 4 Department of Paediatric, Faculty of Medicine, Public Health and Nursing, Universitas Gadjah Mada/Dr Sardjito Hospital, Yogyakarta, Indonesia

**Keywords:** healthcare facilities, risk, nosocomial, prevention, workforce

## Abstract

**SETTING:**

Healthcare workers (HCWs) are at an increased risk of TB worldwide. Individual knowledge and attitudes may influence HCW behaviour, and subsequently, TB risk. Indonesia has the second highest case-load globally.

**OBJECTIVE:**

To measure TB knowledge and attitudes among a subsection of HCWs in Yogyakarta, Indonesia, and to explore factors associated with knowledge.

**DESIGN:**

A cross-sectional study using an online survey targeting all HCW staff was conducted among HCWs from four pre-selected healthcare facilities in Yogyakarta. Descriptive analysis and a multivariable linear regression were undertaken.

**RESULTS:**

Of 792 HCWs, 290 (37%) completed the survey; 64% (*n* = 185) were medical staff, 33% (*n* = 95) reported previously being tested for active TB and 8% (*n* = 24) for latent TB. The mean knowledge score was 7.2/11 (SD 1.5): this was higher among medical staff and those with university education (average score increase: 0.53, 95% CI 0.15 to 0.90; and 0.38, 95% CI 0.01 to 0.74, respectively). Participants agreed that free access to TB screening (93%) and treatment (93%) should be available, and 57% of medical and 77% of non-medical staff would take preventive therapy if eligible.

**CONCLUSION:**

Participants had practical understanding of TB; however, gaps were identified in knowledge about TB disease progression and prevention. Prevention programmes were viewed positively. We suggest further TB education and engagement programmes for HCWs.

## Abstract (fr)

**CONTEXTE:**

Les travailleurs de la santé (HCW) sont exposés à un risque accru de TB dans le monde entier. Les connaissances et les attitudes individuelles peuvent influencer le comportement des HCW et, par conséquent, le risque de TB. L’Indonésie a le deuxième plus grand nombre de cas dans le monde.

**OBJECTIF:**

Mesurer les connaissances et les attitudes à l’égard de la TB parmi un sous-groupe de HCW à Yogyakarta, en Indonésie, et explorer les facteurs associés aux connaissances de la TB.

**MÉTHODE:**

Une étude transversale a été menée à l’aide d’un sondage en ligne ciblant tous les HCW de quatre établissements de santé présélectionnés à Yogyakarta. Une analyse descriptive et une régression linéaire multivariable ont été effectuées.

**RÉSULTATS:**

Sur 792 HCW, 290 (37%) ont répondu à l’enquête ; 62% (*n* = 181) étaient des membres du personnel médical, 33% (*n* = 95) ont déclaré avoir déjà été testés pour la TB active et 8% (*n* = 24) pour la TB latente. Le score moyen de connaissances était de 7,2/11 (SD 1,5) : il était plus élevé parmi le personnel médical et les personnes ayant une formation universitaire (augmentation moyenne du score : 0,53 ; IC 95% 0,11–0,93 et 0,38 ; IC 95% 0,01–0,74, respectivement). Les participants étaient d’accord pour dire que l’accès au dépistage (93%) et au traitement (93%) de la TB devrait être gratuit, et 57% du personnel médical et 77% du personnel non médical suivraient un traitement préventif s’ils étaient éligibles.

**CONCLUSION:**

Les participants avaient une compréhension pratique de la TB ; cependant, des lacunes ont été identifiées dans les connaissances sur la progression de la maladie et la prévention de la TB. Les programmes de prévention ont été perçus positivement. Nous suggérons d’autres programmes d’éducation et d’engagement sur la TB pour les HCW.

TB is a public health crisis, with an estimated 1.4 million people dying of TB globally in 2019.^1^ The WHO End TB Strategy sets ambitious goals for achieving TB elimination by 2035. Pillars one and two of the End TB Strategy are dependent on a well-resourced, healthy, knowledgeable, supportive and skilled health-care workforce.^2^

Healthcare workers (HCWs), defined by the WHO as “all those in the health sector and other sectors, whose main activities are aimed at improving health, including health service providers and health management and support workers”, are at an increased risk of contracting TB compared to the general population due to potential exposure to infected patients within their workplace.^3^–^5^ Risk of HCWs contracting TB is higher in low- and middle-income countries (LMICs), where exposure to infection is more likely and infection control practices may be inconsistent or inadequate.^6^ Reported estimates of TB infection (TBI) and active TB in HCWs vary widely in LMICs. A recent systematic review found that TBI prevalence based on positive tuberculin skin test (TST) results ranged from 14% to 98% (mean 49%) in LMICs, with higher prevalence seen in higher-burden settings (⩾300 per 100,000).^7^ Others have reported an annual TBI incidence between 0.5% and 14.3% and an incidence of active disease ranging from 69 to 5,780/100,000.^6^,^8^

Individual HCW knowledge and attitudes influence their behaviour and risk of contracting TB.^5^,^8^ The burden of TB in HCWs highlights the fact that occupational TB and nosocomial TB transmission could undermine TB elimination, and emphasises the need for further research, policy and programming.

Indonesia has the second highest TB caseload worldwide, and an annual disease incidence of 312/100,000.^1^ In Indonesia, reported rates of TBI in HCWs range from 24% in primary healthcare centres (PHCs) to 77% in tertiary hospitals, however there is limited information specific to Yogyakarta, or of knowledge and attitudes of HCWs towards TB and TB preventive screening and treatment.^9^,^10^ This study aimed to assess the knowledge of and attitudes towards TB, infection, risk and preventive measures among HCWs in Yogyakarta, Indonesia.

## STUDY POPULATION, DESIGN AND METHODS

### Study setting

The Indonesian health system has multiple levels (national, provincial and district) and a mix of public and private providers.^11^ District-level public and private hospitals and PHCs, known as *Puskesmas* (*pusat kesehatan masyarakat*), provide TB services such as case detection, diagnosis and treatment.^11^ In 2015, notifications from PHCs comprised 71% of all case notifications in Indonesia, and in previous years, private hospital contributed the majority of notifications.^12^

### Study design, sample size, selection and participants

The cross-sectional study recruited participants from four pre-selected facilities. Selection of health facilities was targeted based on the respective sub-district’s involvement in a TB Reach-funded active case-finding programme.^13^ The involvement of the selected sub-districts (one rural and one urban) was based on low case detection rates, provincial and district health office consultation, and operational challenges, including low coverage of testing, under-diagnosis in children and near absence of active case-finding, contact tracing and provision of preventive therapy. All health facilities delivering TB care in these sub-districts were recruited, which included one private hospital treating 100–120 TB patients per year in 2018 and 2019, and three PHCs treating 4–20 TB patients per year in 2018 and 2019.

The target population for this study was all staff aged ⩾17 years at the healthcare facilities, including medical staff and non-medical staff such as management and support workers.^5^ There were 792 staff across the four facilities: 44, 62 and 73 staff at PHCs and 613 at the private hospital.

### Data collection

The knowledge and attitudes survey (Supplementary Data 1) was created based on previously published surveys, and a questionnaire validated by the STOP TB Partnership.^14^–^16^ The survey was designed in English and translated to Indonesian. It included four sections: informed consent, demographics, knowledge, and attitudes. The first section collected informed consent, which was required to continue the survey. All data collected were anonymous and demographic information was collected as broad categories to minimise participant identifiability. Demographic questions included age category, sex, occupation, length of time working in the health system and TB screening history (*n* = 13). Knowledge questions were multi-choice (*n* = 11); attitude questions used a 5-point Likert scale indicating degree of agreement (*n* = 22).

Data were collected using an electronic REDCap database (Vanderbilt University, Nashville, TN, USA) with validation for 1 week at each facility between December 2020 to February 2021.^17^ Healthcare facility management consented to survey distribution to staff. Information, invitations and a generic electronic survey link was sent in Indonesian by the human resource department to staff using their employee registered phone number, via WhatsApp three times throughout the period of data collection. The survey was not configured to allow participants to return and resume a partially filled survey questionnaire. As the survey strictly preserved anonymity, no controls could be implemented to prevent participants from undertaking the survey more than once; however, a de-duplication process was planned. Participation was voluntary, and participants did not receive incentives for participating.

### Data analysis

For analysis, a total knowledge score for each participant was calculated based on the total answers correct out of 11. Where no answer was given to a question on TB knowledge, this was counted as incorrect. The score was analysed as continuous and reported as a mean.^14^ Attitudes questions were collapsed from five into three categories (agree, neutral or disagree) to capture broader trends, and then compared.

The survey data was exported into Stata v15 (StataCorp, College Station, TX, USA) and de-duplicated. The de-duplication process included determining duplicate entries based on the same answer being selected for a series of not personally identifiable information questions, including favourite musician and movie, workplace, age category, sex and occupation. If entries were considered duplicates, the response with more complete knowledge and attitude answers was included. If duplicates had both sections answered, the first entry was taken based on the survey completion timestamp.

Categorical variables were reported as numbers and proportions. Continuous variables were assessed for normality and reported as either mean and standard deviations (SDs) or median and interquartile ranges (IQRs). To investigate factors associated with an increased knowledge score, demographic and occupational factors were identified using univariable and multivariable linear regression and expressed as a coefficient with 95% confidence interval (95% CI). Variables were included in the multivariable model if *P* < 0.2 in the univariable analyses, but those that asked only for sub-sets of the sample were excluded. Backward selection was employed, and selection stopped when all remaining variables had a *P* < 0.1. The final linear regression model met all underlying assumptions of linearity, normality, and constant variance.

### Ethical approval

Ethical approval was obtained from the Faculty of Medicine, Public Health and Nursing ethics committee at the University of Gadjah Mada, Yogyakarta, Indonesia, and from the Alfred Hospital’s Human Research Ethics Committee, Melbourne, VIC, Australia.

## RESULTS

There were 544 survey responses; the demographic section of the survey was complete in 371 of these responses. After de-duplication, there were 290 responses where the knowledge section was undertaken and 274 where the attitude section was undertaken. Insufficient instructions initially resulted in many survey participants completing only page 1 of the electronic survey (demographic information), so after improvements a further request was distributed, and most identified/removed duplicates were these early responses. Only the de-duplicated 290 survey responses where at least the knowledge section was started are included in analyses, which resulted in a response proportion of 37% (290/792) across the four healthcare facilities.

Response proportions varied by facility: 27% (165/613) in the private hospital, 75% (33/44) in the urban PHC, 45% (33/73) and 95% (59/62) in the rural PHCs. Of the 290 participants, 58% (168/290) were female, 31% (89/290) were aged 40–49 years, and 64% (185/290) were medical staff; the median time working in the health sector was 13 years (6–23). A third (95/290, 33%) of the participants reported previous testing for active TB and 8% (24/290) for TBI. Four participants self-reported TBI (4/290, 1.4%) and 5 reported a past active TB diagnosis (5/290, 1.7%) (Table 1).

**TABLE 1:** Demographic and occupational characteristics of healthcare workers who participated in a TB knowledge and attitudes survey in Yogyakarta healthcare facilities

Characteristic	Responses *n* (%)
Healthcare facility ( *n* = 290)	
Urban PHC	33 (11.4)
Rural PHC I	33 (11.4)
Rural PHC II	59 (20.3)
Urban private hospital	165 (56.9)
Age, years ( *n* = 290)	
17–29	70 (24.1)
30–39	90 (31.0)
40–49	89 (30.7)
⩾50	41 (14.1)
Sex ( *n* = 290)	
Male	122 (42.1)
Female	168 (57.9)
Occupation and category ( *n* = 290)	
Medical	185 (63.8)
Nurse	93 (32.0)
Dentist	6 (2.1)
Laboratory scientists	6 (2.1)
Nutritionist	6 (2.1)
Environmental health	4 (1.4)
Epidemiologist	4 (1.4)
Radiographer	3 (1.0)
Midwife	28 (9.7)
Rehabilitation staff (e.g., physiotherapist)	2 (0.7)
Health promotion nurse	2 (0.7)
Pharmacist and assistants	16 (5.5)
Doctor	14 (4.8)
Psychologist	1 (0.3)
Non-medical	101 (34.8)
Administrative staff	30 (10.3)
Management	8 (2.8)
Community health workers	12 (4.1)
Other non-medical personnel	51 (17.6)
Did not answer	4 (1.4)
Education ( *n* = 290)	
Graduated elementary school	1 (0.3)
Graduated middle school	8 (2.8)
Graduated high school	114 (39.3)
Undergraduate university degree	151 (52.1)
Postgraduate university degree	16 (5.5)
Length of time working in healthcare sector, years, median [IQR] ( *n* = 290)	13 [6–23]
Length of time working in current healthcare facility, years, median [IQR] ( *n* = 280)	10 [3–20]
Have ever worked within TB programme or provided services for TB patients, yes ( *n* = 290)	116 (40.0)
Length of time working within TB programme or with TB patients, years, median [IQR] (*n* = 116)	4 [1–11]
Received training about TB, yes ( *n* = 289)	56 (19.4)
Received training about personal protective equipment, yes (*n* = 288)	220 (76.4)
Have had known non-work contact with a TB patient, yes (*n* = 288)	11 (3.8)
Have been tested for latent TB, yes ( *n* = 290)	24 (8.3)
Diagnosed with latent TB, yes (*n* = 24)	4 (17.0)
Have been tested for active TB, yes ( *n* = 290)	95 (32.8)
Diagnosed with active TB, yes (*n* = 94)	5 (5.0)

PHC = primary healthcare centre; IQR = interquartile range.

### Knowledge

The mean knowledge score among participants was 7.2 (SD 1.5). Most HCWs knew TB was infectious (289/290, 99.7%) and curable (282/290, 97.2%) (Table 2). Lower knowledge was seen for questions related to TB progression from infection to disease (127/290, 43.9%), and prevention mechanisms such as interventions to reduce spread (70/290, 24.1%) and the effectiveness of preventive therapy (171/290, 59.0%). Linear regression (Table 3) showed knowledge was similar by facility. Knowledge was higher for those having a medical occupation (average score increase: 0.53, 95% CI 0.15 to 0.90) or a university degree (average score increase: 0.38, 95% CI 0.01 to 0.74).

**TABLE 2:** Number of correctly answered knowledge questions among HCWs who participated in a TB knowledge and attitudes survey in healthcare facilities, Yogyakarta, Indonesia

Knowledge question/statement	Total (*n* = 290) *n* (%)
TB is infectious	289 (99.7)
TB is curable	282 (97.2)
How is TB spread?	208 (71.7)
Is a N95 mask 100% protective against TB?	194 (66.9)
Do all people who become infected by TB develop symptoms and TB disease?	127 (43.9)
What is the most common symptom of pulmonary TB?	274 (94.5)
TB preventive treatment is the same as TB treatment	194 (67.2)
A person who is infected with TB can be prevented from becoming sick with TB by undergoing TB preventive therapy	171 (59.0)
Healthcare staff are more at risk of contracting TB than the general community	193 (66.6)
Which people are at an increased risk of developing TB disease?	71 (24.5)
What interventions can reduce spread of TB in a healthcare facility?	70 (24.1)

**TABLE 3:** Associations between HCW demographic and occupational characteristics and TB knowledge score among HCWs who participated in a TB knowledge and attitudes survey in healthcare facilities, Yogyakarta, Indonesia

Characteristics	Knowledge score Mean ± SD	Coefficient (95% CI)	Adjusted coefficient (95% CI)
Healthcare facility			
Urban PHC	7.3 ± 1.5	0.13 (−0.42 to 0.69)	
Rural PHC I	7.1 ± 1.3	−0.18 (−0.57 to 0.54)	
Rural PHC II	7.1 ± 1.2	0.00 (−0.44 to 0.44)	
Urban private hospital	7.1 ± 1.6	Reference	
Location of healthcare facility			
Rural	7.1 ± 1.2	Reference	
Urban	7.2 ± 1.6	0.03 (−0.33 to 0.43)	
Type of healthcare facility			
Hospital	7.1 ± 1.6	Reference	
PHC	7.2 ± 1.3	0.03 (−0.31 to 0.38)	
Age, years			
17–29	7.0 ± 1.5	Reference	
30–39	7.2 ± 1.4	0.14 (−0.32 to 0.60)	
40–49	7.3 ± 1.6	0.28 (−0.19 to 0.74)	
⩾50	7.1 ± 1.4	0.06 (−0.51 to 0.63)	
Sex			
Male	7.0 ± 1.5	Reference	
Female	7.2 ± 1.5	0.18 (−0.17 to 0.52)	
Occupation category			
Non-medical	6.7 ± 1.4	Reference	Reference
Medical	7.4 ± 1.4	0.67 (0.32 to 1.01)	0.53 (0.15 to 0.90)
Education			
High school certificate or less	6.8 ± 1.3	Reference	Reference
University degree	7.4 ± 1.5	0.63 (0.30 to 0.97)	0.38 (0.01 to 0.74)
Length of time working in healthcare sector		0.01 (−0.01 to 0.03)	
Length of time working in current healthcare facility		0.00 (−0.02 to 0.02)	
Work within TB programme or with TB patients			
No	7.0 ± 1.4	Reference	
Yes	7.4 ± 1.5	0.43 (0.08 to 0.77)	
Received training about TB			
No	7.1 ± 1.5	Reference	
Yes	7.4 ± 1.5	0.33 (−0.10 to 0.75)	
Received training about PPE			
No	6.9 ± 1.6	Reference	Reference
Yes	7.2 ± 1.4	0.29 (−0.11 to 0.69)	0.36 (−0.03 to 0.75)
Have had close non-work contact with a TB patient			
No	7.1 ± 1.5	Reference	
Yes	7.3 ± 0.9	0.14 (−0.75 to 1.03)	
Have been tested for latent TB			
No	7.1 ± 1.5	Reference	Reference
Yes	7.7 ± 1.6	0.57 (−0.05 to 1.18)	0.51 (−0.08 to 1.10)
Have been tested for active TB			
No	7.1 ± 1.5	Reference	
Yes	7.3 ± 1.4	0.15 (−0.21 to 0.51)	

HCW = healthcare worker; SD = standard deviation; CI = confidence interval; PHC = primary healthcare centre; PPE = personal protective equipment.

### Attitudes

Among the 274 participants who completed the attitudes section, most participants believed TB patients have adequate support through their diagnosis (92% agreement) and treatment (84% agreement) (Table 4). A majority disagreed that patients are shunned by their families and communities (73%) or should quit their jobs to prevent transmission (66%). A large proportion disagreed with stigma-related statements such as HCWs are nervous about treating TB patients (68%), avoid them (75%) and that infection is their own fault (74%). Related to HCW risk, most participants believed HCWs are at risk of contracting TB at healthcare facilities (75% agreement), but their facilities do what they can to prevent this (80% agreement). Most participants agreed with the statements supporting implementation of free access to TB screening (93%) and treatment (93%). Non-medical and medical staff expressed similar attitudes for many of the statements (Table 5). However, attitudes to the risk of contracting TB at healthcare facilities (60% vs. 83%) and preference for getting tested and treated at a different healthcare facility (39% vs. 21%) differed. Furthermore, non-medical staff were more willing to take preventive therapy if eligible (77% vs. 57%).

**TABLE 4:** Attitudes about TB among HCWs who participated in a TB knowledge and attitudes survey in healthcare facilities, Yogyakarta, Indonesia

	Agree *n* (%)	Neutral *n* (%)	Disagree *n* (%)
Community awareness and stigma			
There is widespread awareness about TB and TB symptoms in communities (collective view)*	158 (57.9)^†^	55 (20.1)	60 (22.0)
TB patients are supported by their families and communities through their treatment	231 (84.3)^†^	26 (9.5)	17 (6.2)
People who are diagnosed with TB are shunned by their families and communities	41 (15.0)	33 (12.0)	200 (73.0)^†^
I feel stigmatised because my work involves interacting with people with or who have had TB*	22 (8.1)	80 (29.4)	170 (62.5)^†^
If I was sick, I would prefer to get tested and treated at a different healthcare facility*	73 (26.8)	51 (18.8)	148 (54.4)^†^
I would feel scared and ashamed if I was diagnosed with TB*	62 (22.7)	54 (19.8)	157 (57.5)^†^
Perceptions of TB and people with TB			
People with TB should quit their job to prevent further transmission even if they are on treatment	43 (15.7)	50 (18.2)	181 (66.1)^†^
Some HCWs are nervous about treating TB patients*	46 (16.8)	41 (15.0)	186 (68.1)^†^
Some HCWs don’t like helping TB patients*	27 (10.0)	42 (15.6)	201 (74.4)^†^
Some HCWs stay away from TB patients	37 (13.5)	31 (11.3)	206 (75.2)^†^
Some HCWs think developing TB is the person’s own fault	33 (12.0)	38 (13.9)	203 (74.1)^†^
Some HCWs think taking TB treatment should be forced if necessary*	175 (64.1)	42 (15.4)	56 (20.5)^†^
Perceived risk as a HCW			
If HCWs wear a mask at work, they will not contract TB*	185 (68.0)^†^	40 (14.7)	47 (17.3)
HCWs are at risk of contracting TB at the healthcare facility they work*	205 (75.4)	41 (15.1)	26 (9.6)^†^
I feel unsafe at work because I feel that I am at risk of catching TB*	33 (12.1)	68 (25.0)	171 (62.9)^†^
I feel like my workplace does everything it can to protect me against contracting TB*	219 (80.2)^†^	42 (15.4)	12 (4.4)
Health system capacity			
Patients who are diagnosed with TB receive adequate support through their diagnosis*	251 (91.9)^†^	9 (3.3)	13 (4.8)
Healthcare facilities in Yogyakarta have enough staff to treat all TB patients*	177 (64.8)^†^	54 (19.8)	42 (15.4)
TB prevention			
Facilities should provide HCWs with free annual screening for TB infection and disease*	251 (92.6)^†^	10 (3.7)	10 (3.7)
These screenings should be done during work time and hours*	215 (79.0)^†^	37 (13.6)	20 (7.4)
Healthcare facilities should provide free treatment and preventive therapy if their HCWs develop TB*	253 (93.4)^†^	8 (3.0)	10 (3.7)
If I do not have TB disease but I have a risk of developing TB disease due to contact with TB patients at work, I would agree to take TB preventive therapy*	173 (63.6)^†^	51 (18.8)	48 (17.6)

* Not all respondents answered each question (numbers range from 271 to 273 out of 274).

^†^ Indicates a positive attitude towards the question or statement.

HCW = healthcare worker.

**TABLE 5:** TB attitudes by percentage of agreement among medical and non-medical HCWs, who participated in a TB knowledge and attitudes survey in healthcare facilities, Yogyakarta, Indonesia

	Non-medical (*n* = 94)* *n* (%)	Medical (*n* = 176)* *n* (%)
Community awareness and stigma		
There is widespread awareness about TB and TB symptoms in communities^†^	52 (55.9)^‡^	103 (58.5)^‡^
TB patients are supported by their families and communities through their treatment	79 (84.0)^‡^	149 (84.7)^‡^
People who are diagnosed with TB are shunned by their families and communities	13 (13.8)	28 (15.9)
I feel stigmatised because my work involves interacting with people with or who have had TB^†^	12 (13.0)	9 (5.1)
If I was sick, I would prefer to get tested and treated at a different healthcare facility^†^	36 (39.1)	36 (20.5)
I would feel scared and ashamed if I was diagnosed with TB^†^	24 (25.8)	37 (21.0)
Perceptions of TB and people with TB		
People with TB should quit their job to prevent further transmission even if they are on treatment	22 (23.4)	21 (11.9)
Some HCWs are nervous about treating TB patients^†^	14 (14.9)	30 (17.1)
Some HCWs don’t like helping TB patients^†^	6 (6.4)	21 (12.2)
Some HCWs stay away from TB patients	7 (7.4)	29 (16.5)
Some HCWs think developing TB is the person’s own fault	16 (17.0)	16 (9.1)
Some HCWs think taking TB treatment should be forced if necessary^†^	64 (68.8)	109 (61.9)
Perceived risk as a HCW		
If HCWs wear a mask at work, they will not contract TB^†^	63 (67.7)^‡^	119 (68.0)^‡^
HCWs are at risk of contracting TB at the healthcare facility they work at^†^	56 (60.2)	146 (83.4)
I feel unsafe at work because I feel that I am at risk of catching TB^†^	14 (15.1)	18 (10.3)
I feel like my workplace does everything it can to protect me against contracting TB^†^	73 (78.5)^‡^	142 (80.7)^‡^
Health system capacity		
Patients who are diagnosed with TB receive adequate support through their diagnosis^†^	83 (89.2)^‡^	164 (93.2)^‡^
Healthcare facilities in Yogyakarta have enough staff to treat all TB patients^†^	66 (71.0)^‡^	108 (61.4)^‡^
TB prevention		
Facilities should provide HCWs with free annual screening for TB infection and disease^†^	84 (90.3)^‡^	164 (94.3)^‡^
These screenings should be done during work time and hours^†^	65 (70.7)^‡^	147 (83.5)^‡^
Healthcare facilities should provide free treatment and preventive therapy if their HCWs develop TB^†^	82 (89.1)^‡^	167 (95.4)^‡^
If I do not have TB disease but I have a risk of developing TB disease due to contact with TB patients at my work, I would agree to take TB preventive therapy^†^	72 (77.4)^‡^	100 (57.1)^‡^

* 4/270 participants are not included as their occupations were unknown.

^†^ Not all respondents answered each question (numbers range from 266 to 269 out of 270).

^‡^ Indicates a positive attitude towards the question or statement.

HCW = healthcare worker.

## DISCUSSION

This cross-sectional study aimed to assess the knowledge and attitudes towards TB among HCWs across four different healthcare facilities in two rural and urban sub-districts of Yogyakarta, Indonesia. We found gaps in knowledge among study participants about TB infection, disease progression, prevention and treatment. Higher TB knowledge was significantly associated with having a medical occupation or university degree, although the increase in knowledge score was relatively small. There were positive attitudes towards the provision of TB prevention in health-care facilities.

The identified knowledge gaps in our study were mostly related to TB prevention, consistent with knowledge of HCWs in other high TB incidence settings. A survey in Vietnam found the lowest TB knowledge was around identification of high-risk groups, prevention of infection and progression to disease.^18^ Similarly, only 55% of HCWs in Peru knew not all people infected with TB develop symptoms.^14^ In Brazil, a survey found that 60% of auxiliary HCWs could not clearly distinguish active TB from TBI and did not know how to diagnose TBI, while half did not know how to prevent progression to active TB.^19^ In Indonesia, medically trained HCWs at a public hospital in Bandung had knowledge gaps in healthcare facility-level and individual-level infection prevention and control (IPC) measures.^20^

Level of TB knowledge was associated with having a medical occupation and university degree, and although the detected difference was small and may not be meaningful in their work or risk behaviour, studies in other high-incidence settings have found similar associations.^18^,^20^–^22^ Current post-qualification training for TB in Indonesia is limited to medical staff and is largely focused on TB screening for active disease, diagnosis and treatment. This may explain the gap in basic knowledge among all participants in TBI and TB preventive measures such as preventive therapy and screening, which are a relatively new concept nationally.

During the study period, healthcare facilities in Indonesia did not actively screen for active or latent TB, or provide preventive therapy for their staff, although they had been listed as high-risk in the 2016–2020 National Strategic Plan and were expected to be provided with these services.^12^ This is suggested to be due to the lack of healthcare facility awareness of this recent recommendation, budget restrictions and the lack of a national guideline for HCW screening. Our study found that despite this gap in the TB programme, most participants have positive attitudes to TB prevention interventions. However, medical staff, who had higher TB knowledge, were less likely to want to undergo preventive therapy. Similar studies in other countries have found positive HCW attitudes towards TB prevention for HCWs. A study among Australian hospital staff found that, although positive attitudes towards preventive therapy existed, knowledge of TBI was low, as was the uptake of preventive therapy.^23^ A study among US HCWs found barriers to TB screening included a perception that preventive therapy was harmful, and a distrust of the facilities employees health programmes.^24^ These studies suggest more than just knowledge and positive attitudes towards screening and treatment are required to increase TB preventive therapy uptake by healthcare staff.

This study had several limitations. First, the cross-sectional design and target population of all staff at purposively selected study sites meant that the knowledge and attitudes of HCWs may not be generalisable to the district and provincial level. Second, desirability bias may have skewed participation and responses. We attempted to minimise this by having the human resource department share the survey link and information reiterating that the survey was voluntary, anonymous and the information collected was de-identified and confidential. Third, the overall response rate was only 37%, and varied by facility. Fourth, it did not assess HCW practice or behaviour, particularly related to infection risk, to further explore the association with knowledge and attitudes.

Moreover, there is a risk of multiple responses in the anonymous survey with a non-unique access link. To limit this, we included questions about recreational preferences that aimed to generate unique combinations of information to facilitate de-duplication without being personally identifiable, and create a de-duplication algorithm. However, there is a chance that the dataset may contain a small number of instances of an individual completing the survey more than once. The mechanism of distribution (electronic) may have restricted some HCWs taking part in the survey, biasing the results towards a particular demographic. Finally, although delivered in Indonesian, some participants may have misunderstood some questions. We attempted to ensure clarity by basing the survey on validated templates or previous published surveys, with translation and back translation of the survey by two translators, and piloting the survey among several Indonesian HCWs working within our programme. Strengths of this study include a diversity of occupations of HCWs represented and robust data collection, allowing for associations between knowledge and demographic and occupational characteristics to be determined.

Our study has identified areas for further TB education and engagement among HCWs in these facilities, and those that may be more broadly beneficial in Yogyakarta, Indonesia. Given HCWs are at increased risk of contracting TB and that their individual knowledge and attitudes may influence their behaviour, practice, and subsequently their TB infection risk, it is important to reduce these knowledge gaps, engage this population and promote positive attitudes. Mechanisms for this may include training and education such as post-qualification refresher, or regular training and information sharing for all HCWs, both non-medical and medical. Trainings and education such as the routine NTP training could include modules on TBI, preventive therapy and IPC, be extended to all healthcare facility personnel, not just clinical personnel. Furthermore, the implementation and assessment of preventive measures in healthcare facilities across Indonesia should be prioritised to prevent nosocomial transmission and reduce TB risk in HCWs, including annual staff TB screening, access to preventive therapy and clear national guidelines. This study indicated that the participating HCWs would like these interventions to be prioritised. Further engagement and research into how this could best work for HCWs in Indonesia is needed. Finally, it would be valuable to investigate the association between HCW knowledge and attitudes and their behaviours/practices using a larger representative sample in Yogyakarta, and what this means for their TB risk, to inform interventions to protect HCWs and the communities the healthcare facilities serve.

## CONCLUSION

Indonesian HCWs who participated in this study had practical understanding of TB but there were knowledge gaps surrounding disease progression, TBI and TB prevention. A majority of HCWs had positive attitudes towards prevention programmes. We suggest further training, education and engagement with this population surrounding these identified gaps and prevention programmes.
